# Nanocrystalline hydroxyapatite enriched in selenite and manganese ions: physicochemical and antibacterial properties

**DOI:** 10.1186/s11671-015-0989-x

**Published:** 2015-07-03

**Authors:** Joanna Kolmas, Ewa Groszyk, Urszula Piotrowska

**Affiliations:** Department of Inorganic and Analytical Chemistry, Medical University of Warsaw, Faculty of Pharmacy and Laboratory Medicine, ul. Banacha 1, 02-097 Warsaw, Poland

**Keywords:** Nanocrystalline hydroxyapatite, Selenite, Manganese, Physicochemical properties, Infrared spectroscopy, Powder diffractometry, Nuclear magnetic resonance, Antibacterial properties, Biomaterials, Bioceramics

## Abstract

In this work, we used the co-precipitation method to synthesize hydroxyapatite (Mn-SeO_3_-HA) containing both selenium IV (approximately 3.60 wt.%) and manganese II (approximately 0.29 wt.%). Pure hydroxyapatite (HA), hydroxyapatite-containing manganese (II) ions (Mn-HA), and hydroxyapatite-containing selenite ions alone (SeO_3_-HA), prepared with the same method, were used as reference materials. The structures and physicochemical properties of all the obtained samples were investigated. PXRD studies showed that the obtained materials were homogeneous and consisted of apatite phase. Introducing selenites into the hydroxyapatite crystals considerably affects the size and degree of ordering. Experiments with transmission electron microscopy (TEM) showed that Mn-SeO_3_-HA crystals are very small, needle-like, and tend to form agglomerates. Fourier transform infrared spectroscopy (FT-IR) and solid-state nuclear magnetic resonance (ssNMR) were used to analyze the structure of the obtained material. Preliminary microbiological tests showed that the material demonstrated antibacterial activity against *Staphylococcus aureus,* yet such properties were not confirmed regarding *Escherichia coli.*

**PACS codes:** 61, 76, 81

**Electronic supplementary material:**

The online version of this article (doi:10.1186/s11671-015-0989-x) contains supplementary material, which is available to authorized users.

## Background

Calcium-deficient hydroxyapatite enriched with various ions, i.e., of carbonates, magnesium, sodium, or manganese, is the main inorganic component of mineralized tissues (bone, tooth enamel, dentin, and cementum) [1, 2]. Synthetic hydroxyapatite is one of the most important materials used in bone implant surgery because of its unique properties such as biological activity, biocompatibility, and very good adaptation under in vivo conditions [3, 4]. The primary feature of hydroxyapatite is its ability to be doped with various ions in order to change physical, chemical, and biological properties of apatites [5].

Selenium is an essential trace element in human diet. Recommended daily intake of this mineral nutrient is 55 μg for both men and women [6]. It has been associated with many health benefits in humans and other mammals, such as decreased incidence of cancer, protection against cardiovascular diseases, treating particular muscle disorders, and delaying the onset of AIDS in HIV-positive patients [7, 8]. It has been reported that selenium deficiency can retard growth and change bone metabolism [9]. Selenium is a constituent of selenoproteins and glutathione peroxidase (GPx1 - GPx6), the enzyme that protects cellular membranes against reactive oxygen species (ROS). An increased level of ROS could suppress osteoblastic differentiation of bone marrow stromal cells and contribute to osteoporosis [10]. Selenium also impacts on the induction of cancer cell apoptosis. Selenium-doped hydroxyapatites seem to be favorable materials for treatment of bone cancers such as osteosarcoma or bone metastasis to reduce the probability of recurrence [11–13]. Moreover, it has been proven that selenium may inhibit the activity of *Staphylococcus aureus* and *Pseudomonas aeruginosa* [14]. Its antibacterial mechanism is associated with oxidative stress: selenium promotes the production of ROS, reacting with the bacterial wall or membrane and causing the death of bacteria cells.

Manganese is an important microelement with recommended daily intake of approximately 1–15 mg. It plays an active role as a cofactor for a wide group of enzymes such as superoxide dismutase [15]. It is also necessary for normal development and metabolism of bones and muscles. Manganese takes part in the formation of plasmatic and extracellular proteins, for example collagen, the main structural protein of connective tissues [16]. It is also involved in the synthesis of mucopolysaccharides, which are responsible for cartilage formation [17]. Moreover, manganese induces integrins, which are mediators of cell adhesion [18]. The addition of manganese to hydroxyapatites could improve the bone/implant connection and facilitate bone tissue regeneration. Substitution of manganese may also have an impact on the mechanical properties of the material. Several studies have shown that Mn-doped hydroxyapatite shows higher thermal stability compared with pure hydroxyapatites [19–23].

In this work, we have decided to synthesize hydroxyapatite containing manganese and selenite ions. We have focused on the chemical structure and physicochemical properties of the obtained material. To achieve this goal, we have used various analytical methods such as powder X-ray diffractometry (PXRD), transmission electron microscopy (TEM), wavelength-dispersive X-ray fluorescence (WD-XRF), infrared spectroscopy (FT-IR), solid-state nuclear magnetic resonance (ssNMR), and thermogravimetry (TGA). We have also tested the antibacterial activity of the material on *S. aureus* and *E. coli.*

## Methods

### Preparation of samples

Hydroxyapatite doped with selenite and manganese II ions was prepared with the standard wet method (co-precipitation) using the reagents Ca(NO_3_)_2_ · 4H_2_O, (NH_4_)_2_HPO_4_, Na_2_SeO_3_ · 5H_2_O, and (CH_3_COO)_2_Mn as sources of calcium, phosphorus, selenium, and manganese, respectively. All the reagents were bought from Sigma-Aldrich, Poland. The reagents were weighed out so that the mole ratio of Ca + Mn/P + Se would be ca. 1.67. An aqueous solution containing selenite and phosphate ions was added drop by drop to an aqueous solution containing ions of calcium and manganese. During the synthesis, intensive mixing and slightly increased temperature (40–50 °C) were maintained. After the instillation was finished, the pH of the suspension was set to approximately 9 using concentrated ammonia solution. Intensive mixing and heating was maintained for approximately 2 h, and then the obtained precipitate was left for 24 h to age. Then, the obtained precipitate was filtrated and washed with distilled water multiple times in order to remove excess ammonia and the dissolved reaction products. The precipitate was then dried at 130 °C for 12 h.

The samples of pure hydroxyapatite (HA) and those containing only manganese (II) ions (Mn-HA) or only selenite ions (SeO_3_-HA) were obtained the same way, keeping the same ratio of reagents and the same synthesis conditions.

### Characterization

The crystals of the obtained materials were observed with the use of transmission electron microscopy (JEM 1400; Jeol Co., Japan). For each test, a drop of ethanol suspension of the investigated powder was placed on a Cu mesh covered with a formvar film, then allowed to dry in air and measured under the accelerating voltage of 80 kV. Averaged crystal sizes (in the HA and Mn-HA samples) were calculated from at least 60 randomly selected 2D crystal images using the STATISTICA 64 software (Version 10, StatSoft, Inc. 2011).

The phase composition was tested with the powder diffractometry method using an X’pert Pro diffractometer (Philips). The obtained diffractograms were also used to estimate the lattice parameters. For the calculations, Rietveld method was used (X’pert HighScore Plus software). The crystal size was calculated using Scherrer’s formula [24]:

1 $$ D=\frac{0.94\times \lambda }{\beta_{1/2}\times \cos \theta }, $$

where

*D* is the domain size (crystallite size in nanometers),

*λ* is the wavelength of radiation (in nanometers),

*β*_1/2_ is the peak full width at half maximum (in radians), and

*θ* is the diffraction angle of the corresponding reflex.

Full width at half maximum of the reflex (002) was used to evaluate the crystallinity of the investigated materials in accordance with the following formula [25]:

2 $$ {\chi}_c={\left(\frac{K}{\beta_{(002)}}\right)}^3, $$

where

*χ*_*c*_ is the crystallinity degree corresponding to the fraction of crystalline phase in powder,

*K* is a constant (for a great number of hydroxyapatites, *K* is 0.24),

*β*_(002)_ is the peak (002) full width at half minimum (in degrees).

Elemental analysis was conducted with the wavelength-dispersive X-ray fluorescence (WD-XRF) method after dissolving the samples in HNO_3_ solution (Advant’XP; THERMO ARL).

For comparison, energy-dispersive X-ray spectroscopic microanalysis (EDX, Jeol JEM 1400) was also used to evaluate the content of Ca, P, Mn, and Se. The measurements were repeated six times and then averaged.

In order to measure the water content, thermogravimetric analysis (TGA) was carried out. The measurements were done on TGA Q50 (TA instruments) under nitrogen atmosphere. The temperature range was from the room temperature to 700 °C and heating rate was set as 10 °C/min.

Infrared spectroscopy measurements were done using a Perkin Elmer Spectrum 1000 spectrometer. The transmission technique was applied with the use of KBr tablets. The spectra were obtained within the range of 4000-400 cm^−1^, with 2 cm^−1^ resolution and 30 scans.

The high-resolution ^31^P solid-state nuclear magnetic resonance (ssNMR) spectra were recorded with a Bruker Avance 400 WB spectrometer in a 9.4 T magnetic field. Magic angle spinning (MAS) probe and 4 mm zirconia rotors spun by dry air were used. The ^31^P NMR spectra were recorded using the standard one-pulse-acquire technique (Bloch-decay; BD) under MAS at 7 kHz. Recycle delay and π/2 pulse were optimized at 30 s and 2.8 μs, respectively. For all the measurements, 32 scans were performed. All the obtained spectra were processed using the ACDLabs 10.0 software.

### In vitro antibacterial activity

The antimicrobial activity of the tested materials was determined against gram-positive (*S. aureus* ATCC 25293) and gram-negative (*E. coli* ATCC 2592) bacterial strains. The synthesized hydroxyapatite powders were uniaxially compacted into discs of diameter 13 mm and then heated at 180 °C for 4 h in air. Antibacterial action of hydroxyapatite discs was studied using a log reduction assay [26]. Microbial suspensions of 1.5 × 10^8^ CFU/ml corresponding to 0.5 McFarland density obtained from an overnight culture of bacteria developed on solid media were used. The hydroxyapatite discs and the bacterial suspension were added into sterile test tubes containing 2 ml of tryptic soy broth to make the final concentration of the bacterial cells approximately 2 × 10^7^ CFU/ml. Undoped HA was used as a control. A negative control which contained 2 × 10^7^ CFU/ml was also prepared for comparison. The tubes were incubated at 37 °C for 24 h. Serial dilutions of the bacteria were plated onto nutrient agar in triplicate and incubated at 37 °C for 18 h. The colony formation was then examined and counted.

## Results and discussion

### Transmission electron microscopy, powder X-ray diffractometry, and elemental and thermogravimetric analysis

**Fig. 1:**
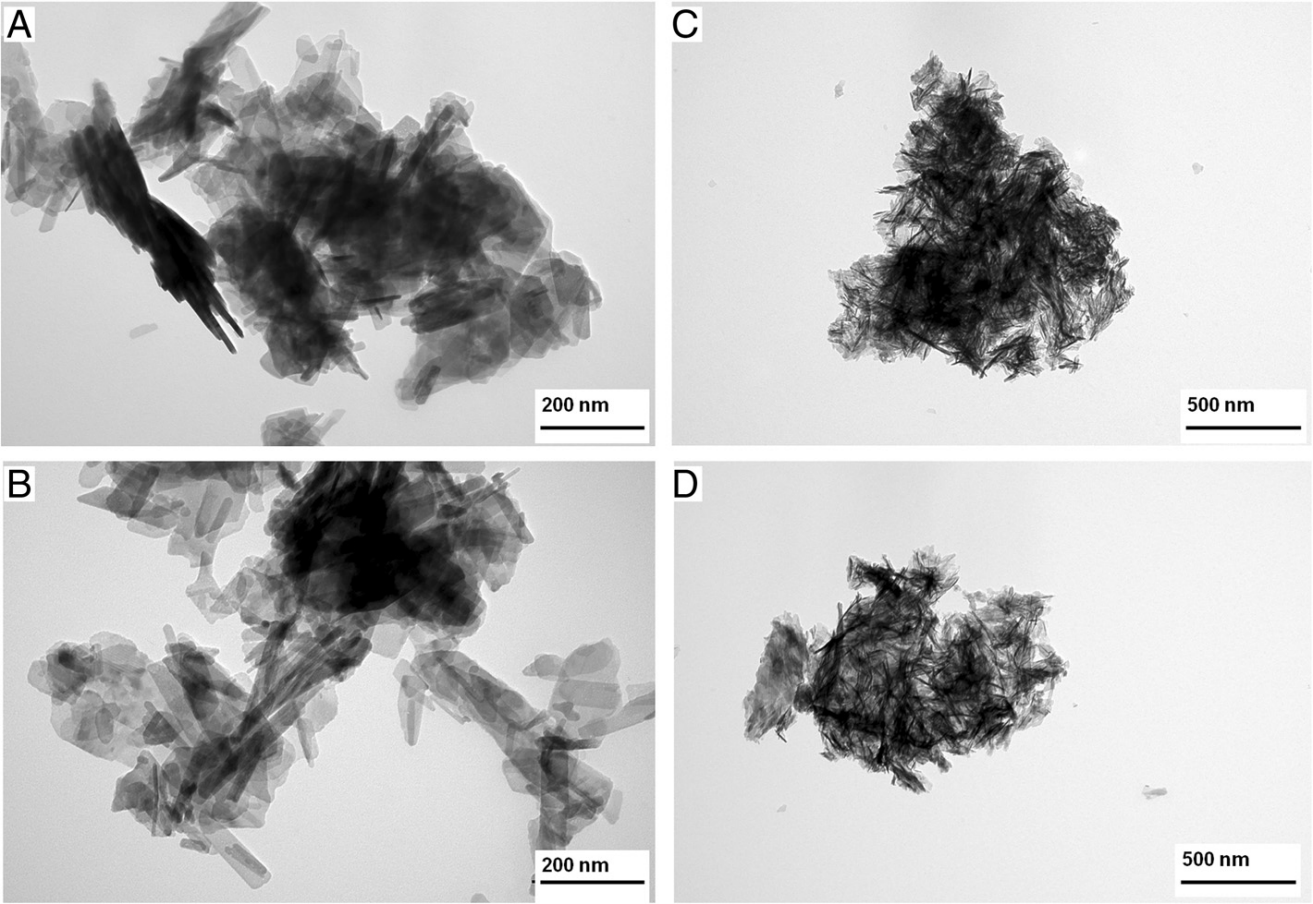
TEM images of the analyzed samples: HA (**a**); Mn-HA (**b**); SeO_3_-HA (**c**) and Mn-SeO_3_-HA (**d**)

The crystals’ morphology and dimensions were evaluated on the basis of TEM images (Fig. 1a–d). In all the samples, crystals had the tendency to form compact agglomerates. The HA and Mn-HA samples had crystals with similar sizes and shapes: the shape was elongated, rod-like, and the mean length and width of crystals did not exceed 150 and 40 nm, respectively. The crystals in the samples of SeO_3_-HA and Mn-SeO_3_-HA were much smaller, and their shape more needle-like. It can be concluded that the presence of manganese ions does not significantly influence the size or morphology of hydroxyapatite crystals, whereas selenite ions make the crystals smaller and change their shape. As it was reported in [11], selenites significantly affect the size of hydroxyapatite crystals. The thermochemical radius of the selenite ions (0.239 nm) is very similar to the radius of the phosphate ions (0.238 nm). However, selenites have a flat trigonal pyramidal shape whereas phosphates are tetrahedral. It is also important to note that substitution of one divalent selenite ion in place of one trivalent orthophosphate results in a simultaneous release of one calcium cation and one hydroxyl anion. These changes may have an influence on the morphology and the size of HA crystals.

Unfortunately, the strong tendency to form clusters (agglomerates) made it impossible to evaluate the sizes of single crystallites in the samples of SeO_3_-HA and Mn-SeO_3_-HA.

**Fig. 2:**
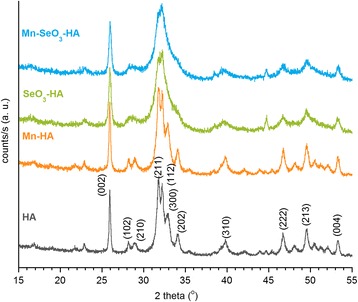
XRD patterns of the analyzed samples

Powder diffractograms of the investigated materials are shown in Fig. 2. Each of the obtained diffractograms corresponds to the standard diffractogram of hydroxyapatite ICDD (no. 9432). The obtained results show that the samples are homogeneous and do not include any other crystal phase. It must be noted, however, that the reflexes of the HA and Mn-HA diffractograms are much narrower and better resolved than the reflexes of the diffractograms of the SeO_3_-HA and Mn-SeO_3_-HA samples. This may confirm the results obtained from TEM, indicating that samples containing selenites have much smaller crystallites, and suggest poorer organization of the crystalline structure [27].

The size of crystallites was calculated using the Scherrer’s formula (see “Methods” section). For the HA and Mn-HA samples, it was approximately 23 ± 5 nm; and for the SeO_3_-HA and Mn-SeO_3_-HA samples, it was approximately 12 ± 3 nm (see Table 1). The crystallinity indices determined according to [25] decrease in the following order: HA(1.79) > Mn-HA(1.58) > > SeO_3_-HA(0.38) > Mn-SeO_3_-HA(0.33). The obtained values show that introducing manganese ions into the structure slightly affects the crystallinity of the hydroxyapatite, while the substitution of selenium IV ions significantly decreases the crystal perfection of the material.

**Table 1:** Various parameters of the studied samples

	HA	Mn-HA	SeO_3_-HA	Mn-SeO_3_-HA
Cell parameters (Å)	*a* = 9.424(3)	*a* = 9.428(4)	*a* = 9.444(4)	*a* = 9.443(3)
	*c* = 6.876(2)	*c* = 6.876(3)	*c* = 6.854(3)	*c* = 6.863(4)
Crystallinity index (%)	1.79	1.58	0.38	0.33
Crystal size (nm)	24 ± 5	23 ± 4	13 ± 3	12 ± 3
Water content (wt.%)	5.2 ± 0.2	5.4 ± 0.3	7.6 ± 0.3	7.3 ± 0.2
Se content (wt.%)	-	-	3.71 ± 0.043	3.60 ± 0.033
Mn content (wt.%)	-	0.28 ± 0.02	-	0.29 ± 0.02
(Ca + Mn)/(P + Se)^a^	1.62 ± 0.03	1.59 ± 0.02	1.53 ± 0.03	1.52 ± 0.02
(Ca + Mn)/(P + Se)^b^	1.65 ± 0.06	1.64 ± 0.07	1.57 ± 0.07	1.56 ± 0.07

The unit cell parameters *a* and *c* (Å), crystallinity index [25], and crystal size (along *c* axis) were calculated from the PXRD diffractograms. Chemical composition of the samples was studied using WD-XRF analyses

^a^Calculated from WD-XRF data

^b^Calculated from EDS data

The lattice parameters of the investigated crystal materials were evaluated on the basis of powder diffractograms (see Table 1). The dimensions of the unit cell of the Mn-SeO_3_-HA sample were increased along axis *a* and decreased along axis *c*. From the data presented in Table 1, it can be concluded that the presence of selenite ions determines the parameters of the unit cell more than does the presence of manganese ions.

Elemental analysis was conducted for all the studied samples. Two techniques—WD-XRF and EDS—were used. On the basis of the obtained results, mole ratios of Ca + Mn/P + Se were calculated (see Table 1). The results indicate that in all the samples, the calcium content was reduced in relation to the phosphorus content. This is probably associated with the fact that samples synthesized with the wet method and dried at low temperatures may contain small amounts of acidic phosphates and carbonates; therefore, so as to maintain the charge balance, calcium ions and hydroxyl ions are partially removed from the crystals [28]. The content of manganese in the Mn-HA and Mn-SeO_3_-HA samples, just like the content of selenium in the SeO_3_-HA and Mn-SeO_3_-HA samples, was close to the expected value. This proves the effectiveness of the introduction of both ions into the hydroxyapatite structure.

TGA data of the obtained hydroxyapatites showed the decrease of weight below 200 °C which corresponds to loss of water adsorbed on the crystal surface (see Table 1 and Figure S1 in Additional file 1). The second weight loss in the range 200–400 °C may be assigned to lattice water. The gradual loss of weight between 400 and 700 °C correspond to the decomposition of carbonates, common impurities of hydroxyapatites [29]. The data shows that the total water content is similar in the HA and Mn-HA samples (5.2 and 5.4 %, respectively) and in the SeO_3_-HA and the Mn-SeO_3_-HA samples (7.6 and 7.3 %, respectively). It can be assumed that the higher content of water in selenite-containing samples is due to their smaller crystal size and the high tendency to agglomerate.

### FT-IR spectroscopy

**Fig. 3:**
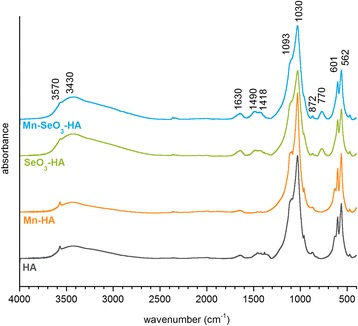
FT-IR transmission spectra of the analyzed samples

Figure 3 presents the FT-IR spectra of the obtained materials. In all the spectra, the 1200–900 cm^−1^ region corresponds to *υ*_1_ + *υ*_3_ phosphate vibrations. Within the range of 650–500 cm^−1^, *υ*_4_ phosphate bands occur [30].

A libration band of hydroxyl groups is also visible in the spectra of the HA and Mn-HA samples in this area (at approximately 630 cm^−1^). A small band of stretching vibrations of OH groups is present at approximately 3570 cm^−1^ in all the spectra; in the spectra of the HA and Mn-HA samples, this band is relatively more intensive. This is probably a result of the mechanism of substitution of selenite groups in place of orthophosphate groups and is associated with crystal sizes. Furthermore, it is known from the literature that the number of OH structural groups also depends on the size of hydroxyapatite crystals: the smaller the crystals, the lower the content of OH groups [31]. It should be noted that the investigations of TEM and PXRD showed that the SeO_3_-HA and Mn-SeO_3_-HA materials had very small crystals.

Within the range of 3700–2500 cm^−1^, there is a wide stretching band; and at approximately 1630 cm^−1^, there is a bending band of water. In the spectra of the SeO_3_-HA and Mn-SeO_3_-HA samples, the relative intensity of those bands against the *υ*_4_ band of phosphates is considerably higher than in the HA and Mn-HA samples. This observation is in accordance with the TGA data. The bands in the range of 1500–1400 cm^−1^ and the 872 cm^−1^ band correspond to the vibration of carbonates. These are predominantly type B carbonates. Their presence is probably the consequence of contamination of the reagents and adsorption of carbon dioxide from air. A distinct band at approximately 770 cm^−1^ only occurs in the spectra of samples containing selenium and originates from selenite vibrations [32].

### ^31^P solid-state NMR spectroscopy

**Fig. 4:**
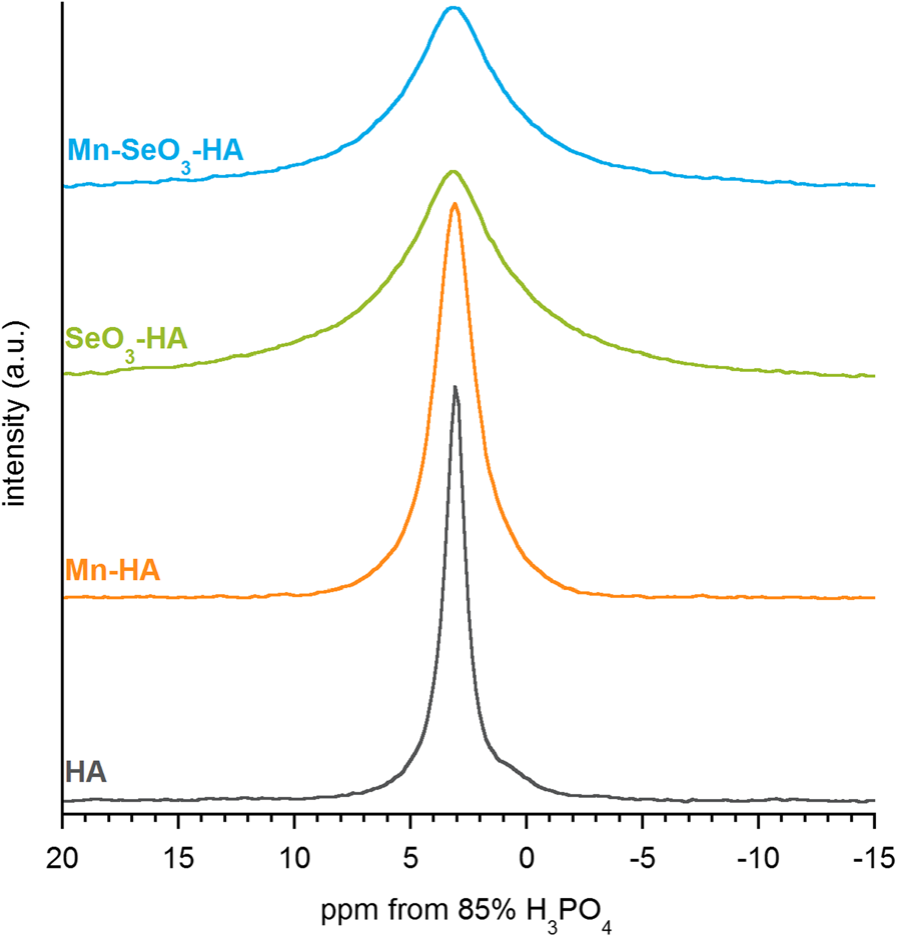
^31^P BD/MAS NMR spectra of the analyzed samples

The spectra of ^31^P BD MAS NMR are presented in Fig. 4. Each spectrum includes one signal at approximately 3.1 ppm, typical for phosphorus-31 nuclei of apatite orthophosphate groups [33]. No signals characteristic of other calcium phosphates, potential “contaminants” of apatite samples, were found. It is important to note that the ^31^P BD NMR lines of substituted hydroxyapatites, especially enriched in selenites, are significantly broader than the line of “pure” hydroxyapatite. Thus, it may be confirmed that incorporation of manganese or/and selenites leads to disorder in the crystalline structure of hydroxyapatite. The signal broadening of the Mn^2+^ ions containing samples (Mn-HA and Mn-SeO_3_-HA) may be also the effect of their paramagnetic properties.

In accordance with previous research done by Pajchel et al. [34], ^31^P BD NMR signals were fitted using two lines: a narrow one, which corresponds to the nuclei of phosphorus-31 originating from inside the crystal, and a broad one, which may be attributed to the nuclei of phosphorus-31 located in the hydrated surface layer. Consider that hydroxyapatite crystals synthesized with the wet method and dried at low temperatures are made up of a well-organized, crystalline core and a surrounding layer (so-called hydrated surface layer), characterized by much poorer organization of the ions located in it. The conducted curve fitting of ^31^P BD NMR signal (see Figure S2 and Table S1 in Additional file 1) shows that the nuclei of phosphorus-31 in the HA and Mn-HA samples are mostly located in the crystal core. It can be concluded that these materials are characterized by greater crystallinity and low contribution of water surface layer, which confirms the results obtained with the TEM, PXRD, and TGA techniques. The contribution of the broad line in the spectra of the SeO_3_-HA and Mn-SeO_3_-HA samples is much greater than in the spectra of the HA and Mn-HA samples.

### In vitro antibacterial activity

**Fig. 5:**
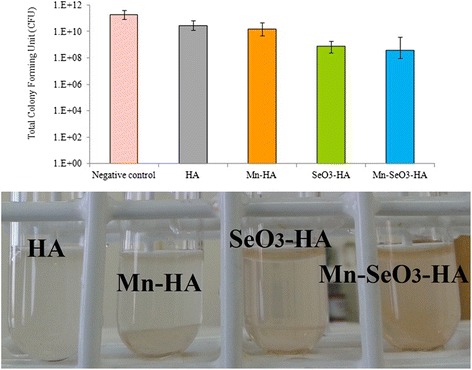
Antibacterials activity of the analyzed samples on *S. aureus*

**Fig. 6:**
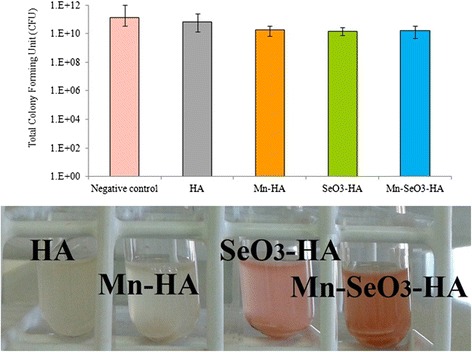
Antibacterials activity of the analyzed samples on *E. coli*

Hydroxyapatite discs were evaluated for their antibacterial activity against gram-positive (*S. aureus*) and gram-negative (*E. coli*) bacteria. The results of the antibacterial activity of different hydroxyapatite discs are presented in Figs. 5 and 6.

*S. aureus*, a virulent pathogen, is currently the most common cause of infections around bone implants in hospitalized patients due to its ability to biofilm formation and documented antibiotic resistance [35]. Selenium, a redox-sensitive element, was found to be an antibacterial agent against *S. aureus*. It can promote the formation of superoxide radicals and provide oxidative stress, resulting in oxidative damage to the bacterial cell walls [14]. Moreover, superoxide radicals are able to inhibit biofilm formation of the *S. aureus* to the solid surface [36, 37]. Figure 5 illustrates the antibacterial activity of different hydroxyapatite discs on *S. aureus* colonies. It should be noted that bacteria will grow rapidly in the nutrient-rich broth, and it has a faster bacterial rate of growth than the bacterial rate of death [26]. Nevertheless, bacterial growth was significantly reduced in the sample with selenite (SeO_3_-HA) compared to the HA and negative control samples (reduction in the number of viable bacteria from 2- to 3-log). Moreover, no significant difference in *S. aureus* growth was observed between the Mn-SeO_3_-HA and SeO_3_-HA discs (Fig. 5). The results of antibacterial tests revealed that the hydroxyapatite discs doped with selenite oxyanions displayed inhibitory activity against *S. aureus* strains. Rodríguez-Valencia et al. [14] have also proved the antibacterial properties of selenite-doped hydroxyapatite. Its antibacterial activity (inhibition of the biofilm formation) on the strains of *S. aureus* was found even at low selenite concentration (0.6 wt.%).

*E. coli* stains were selected for assessment in this study because they are highly capable of reduction of selenite into selenium. Selenium can be afterwards incorporated into proteins as part of the amino acids; selenocysteine or selenomethionine. Depending on the concentration used*,* selenium oxyanions can either stimulate or inhibit *E. coli* growth [38–40]. Figure 6 illustrates the antibacterial activity of different hydroxyapatite discs on *E. coli*. There was no significant difference in the total number of bacteria between the control and the hydroxyapatites disc doped with manganese ions and/or selenite oxyanions (Fig. 6). The SeO_3_-HA and Mn-SeO_3_-HA discs led the culture to turn red, indicating that selenite reduction into selenium (Se^0^) occurred. The results show that 3.5 wt.% selenite concentration did not inhibit growth of test strains. Higher selenite concentration is probably needed to reduce bacterial growth.

## Conclusions

This research involved the synthesis of hydroxyapatite modified with selenite and manganese II ions. Its physicochemical properties were investigated, and its antibacterial activity was preliminarily evaluated. The obtained results can be summarized as follows:

- The obtained material is a nanocrystalline apatite with a hexagonal structure.
- The obtained hydroxyapatite Mn-SeO_3_-HA has crystals with dimensions of approximately 12 ± 3 nm. The crystals demonstrate a strong tendency to form agglomerates.
- The sample of Mn-SeO_3_-HA is homogeneous. Apart from a crystalline core, crystallites have a very complex hydrated surface layer.
- Changes in lattice parameters indicate the introduction of selenite and manganese II ions into the hydroxyapatite structure.
- The obtained material includes approximately 3.6 % weight of selenium and approximately 0.29 % weight of manganese; these values are as expected.
- The water content in the Mn-SeO_3_-HA (7.3 wt.%) and the SeO_3_-HA (7.6 wt.%) samples is higher than in the HA (5.2 wt.%) and the Mn-HA (5.4 wt.%) samples.
- The obtained material is calcium-deficient and has fewer OH structural groups than stoichiometric hydroxyapatite. This probably results from the mechanism of selenium ion substitution and very small crystal sizes.
- Hydroxyapatite enriched with selenite and manganese II ions demonstrates antibacterial activity against *S. aureus* but not against *E. coli.*

The above investigation showed that selenite- and manganese-enriched hydroxyapatite may potentially act as a bone substitute with additional antibacterial properties. Future studies will focus on the cytotoxicity and biocompatibility assays.

## Additional file

Additional file 1: Figure S1. (concerning TGA results), **Figure S2**, and **Table S1** (concerning NMR results).

## Abbreviations

FT-IR spectroscopy: Fourier transform infrared spectroscopy
TEM: transmission electron spectroscopy
ssNMR: solid-state nuclear magnetic resonance spectroscopy
WD-XRF: wavelength-dispersive X-ray fluorescence
EDS: energy-dispersive spectroscopy
PXRD: powder X-ray diffractometry
TGA: thermogravimetry
