# Supplementary material for: Nanocrystalline hydroxyapatite enriched in selenite and manganese ions: physicochemical and antibacterial properties
Source: Nanoscale Res Lett. 2015 Jul 3;10:278. doi: 10.1186/s11671-015-0989-x (PMC4489964; doi:10.1186/s11671-015-0989-x)
Supplement: Additional file 1: Figure S1. — (concerning TGA results), Figure S2, and Table S1 (concerning NMR results). [file 11671_2015_989_MOESM1_ESM.pdf]

SUPPLEMENTARY MATERIALS

Nanoscale Research Letters

Nanocrystalline hydroxyapatite enriched in selenite and manganese ions: physicochemical and antibacterial properties

Joanna Kolmas[1*], Ewa Groszyk[1], Urszula Piotrowska[1]

[1]*Medical University of Warsaw, Faculty of Pharmacy and Laboratory Medicine, Department of Inorganic and Analytical Chemistry, ul. Banacha 1, 02-097 Warsaw, Poland*

E-mail:

J.K.: joanna.kolmas@wum.edu.pl

E.G.: ewa.groszyk@onet.pl

U.P.: piotrowska_urszula@wp.pl

[*]Corresponding author:

Joanna Kolmas

Medical University of Warsaw

Faculty of Pharmacy and Laboratory Medicine

Department of Inorganic and Analytical Chemistry

ul. Banacha 1, 02-097 Warsaw, Poland.

Phone: +48 22 5720755

Fax: +48 22 5720784

E-mail: joanna.kolmas@wum.edu.pl

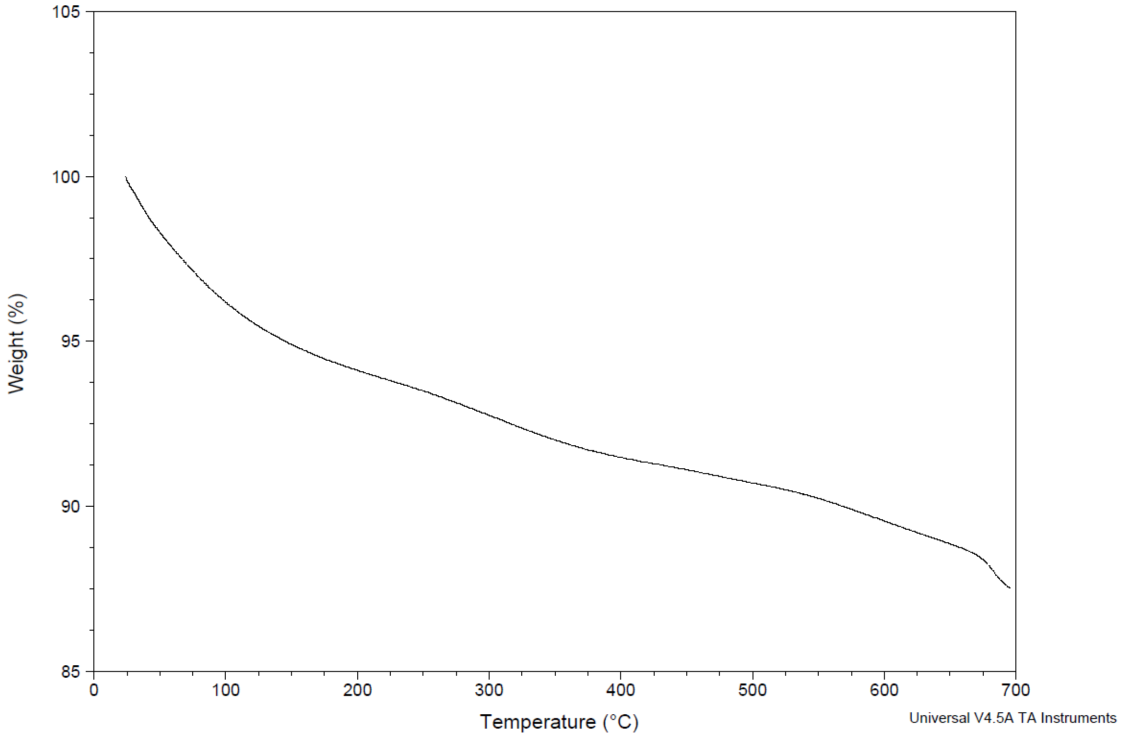

Figure 1S. Thermogravimetric curve of the Mn-SeO_3-HA sample.

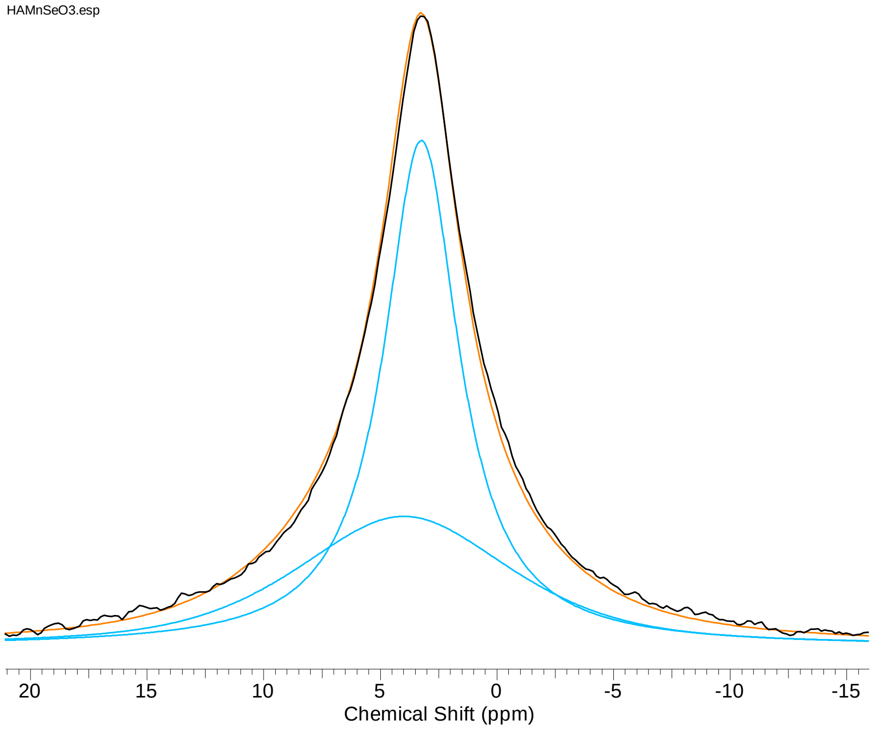

Figure 2S. Representative peak fittings of the ^{31}P BD MAS NMR spectrum (for the Mn-SeO_3-HA sample).

Table 1S. Curve fitting results for the ^{31}P BD and BD NMR spectra (MAS at 7.0 kHz). The peaks have been assigned according to Pajchel et al. [33].

	**HA**	**Mn-HA**	**SeO_3-HA**	**Mn-SeO_3-HA**
**Narrow line**				
Chemical shift (ppm)	3.10	3.13	3.12	3.20
FWHM* (Hz)	176	507	328	630
% of total area	71	79	66	58
**Broad line**				
Chemical shift (ppm)	3.24	3.43	3.30	3.87
FWHM (Hz)	1608	1540	1267	1770
% of total area	29	21	34	42

*FWHM – Full width in a half minimum
